# COVID-19: Progress in diagnostics, therapy and vaccination

**DOI:** 10.7150/thno.47987

**Published:** 2020-06-19

**Authors:** Xue Liu, Chao Liu, Gang Liu, Wenxin Luo, Ningshao Xia

**Affiliations:** 1 State Key Laboratory of Molecular Vaccinology and Molecular Diagnostics & National Institute of Diagnostics and Vaccine Development in Infectious Disease, School of Public Health, Xiamen University, 361102, China.; 2 State Key Laboratory of Molecular Vaccinology and Molecular Diagnostics & Center for Molecular Imaging and Translational Medicine, School of Public Health, Xiamen University, Xiamen, 361102, China.

**Keywords:** COVID-19, SARS-CoV-2, diagnostics, antiviral therapy

## Abstract

Coronavirus disease 2019 (COVID-19) caused by severe acute respiratory syndrome coronavirus 2 (SARS-CoV-2) has recently become a pandemic. As the sudden emergence and rapid spread of SARS-CoV-2 is endangering global health and the economy, the development of strategies to contain the virus's spread are urgently needed. At present, various diagnostic kits to test for SARS-CoV-2 are available for use to initiate appropriate treatment faster and to limit further spread of the virus. Several drugs have demonstrated *in vitro* activity against SARS-CoV-2 or potential clinical benefits. In addition, institutions and companies worldwide are working tirelessly to develop treatments and vaccines against COVID-19. However, no drug or vaccine has yet been specifically approved for COVID-19. Given the urgency of the outbreak, we focus here on recent advances in the diagnostics, treatment, and vaccine development for SARS-CoV-2 infection, helping to guide strategies to address the current COVID-19 pandemic.

## Introduction

The rapid spread of SARS-CoV-2, a novel coronavirus that emerged in December 2019, and the resulting disease, namely, coronavirus disease 2019 (COVID-19), has posed a serious global public health emergency. Whole-genome sequencing results show that the causative agent is a novel coronavirus that was initially named 2019-nCoV by the World Health Organization (WHO) 1-3. COVID-19 was subsequently recommended as the disease name. As the genomic sequence of the new virus is closely related to that of severe acute respiratory syndrome (SARS) coronavirus (SARS-CoV), the International Committee on Taxonomy of Viruses (ICTV) officially designated the virus SARS-CoV-2 4. Patients infected by SARS-CoV-2 manifest a range of symptoms, such as dry cough, fever, headache and dyspnea, and are generally diagnosed with viral pneumonia 5, 6, but asymptomatic infections have also been reported 7, 8.

Coronaviruses, a genus in the *Coronaviridae* family, are enveloped, positive-sense, single-stranded RNA viruses with the largest known genomes (from 25 to 32 kb) among RNA viruses 9, 10. *Coronavirinae* is subdivided into four genera: alpha-, beta-, gamma-, and delta coronaviruses 11. Phylogenetic analysis of coronavirus genomes has revealed that SARS-CoV-2 is a new member of the *Beta coronavirus* genus, which includes SARS-CoV and Middle East respiratory syndrome (MERS) coronavirus (MERS-CoV), as well as other viruses that infect humans and diverse animal species. Among the several coronaviruses that are pathogenic to humans, most are associated with mild clinical symptoms (e.g., HCoV-229E, HCoV-NL63, HCoV-OC43, and HCoV-HKU1) 12, 13, with three notable exceptions: SARS-CoV in November 2002 14-16, MERS-CoV in April 2012 17-19, and, more recently, SARS-CoV-2 causing COVID-19. SARS-CoV provoked a large-scale epidemic beginning in China and involving 37 countries with approximately 8,000 cases and 774 deaths 20; MERS-CoV began in Saudi Arabia and caused a total of 2,519 MERS cases with 866 associated deaths as of the end of January 2020 21.

**Figure 1:**
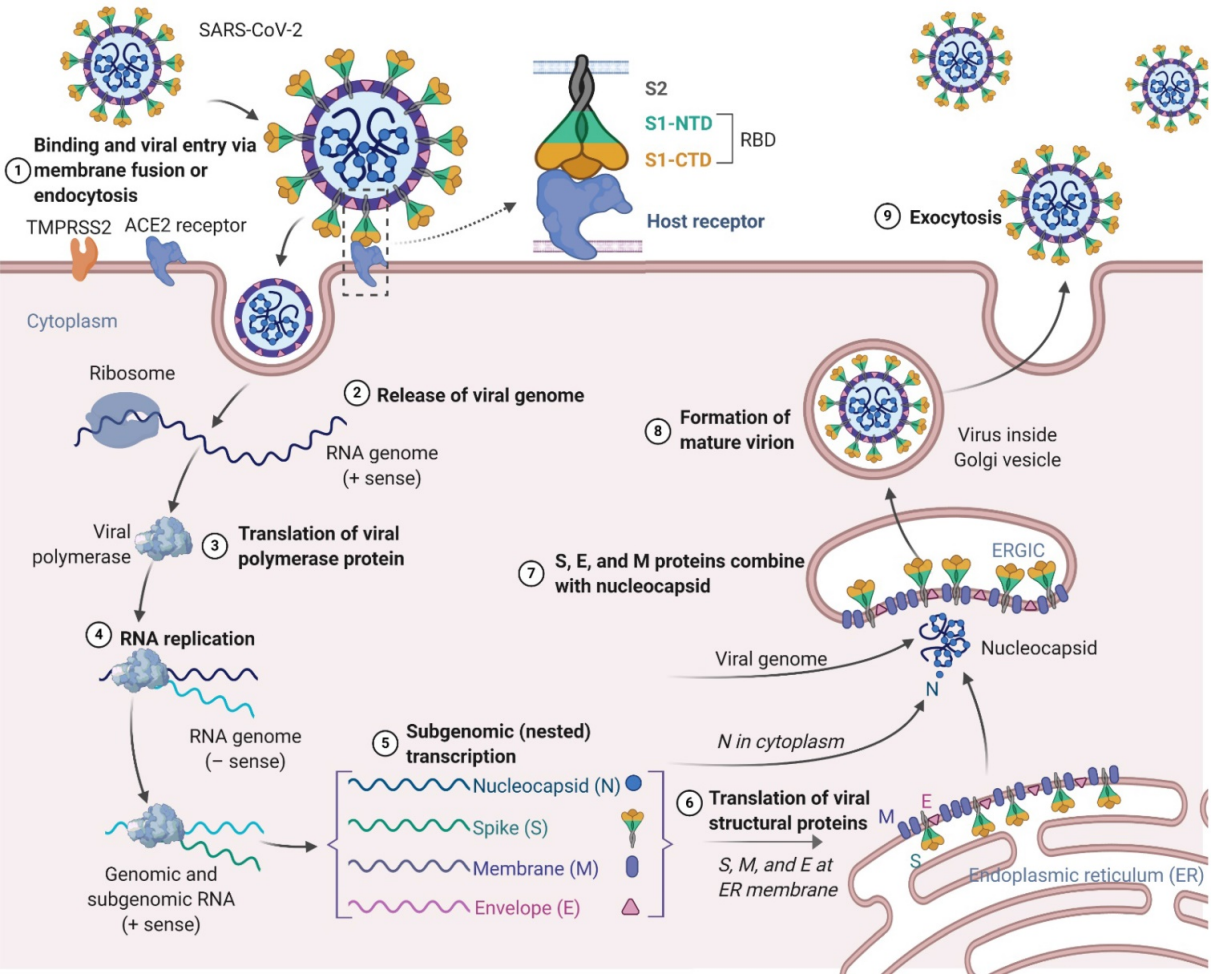
Schematic presentation of the SARS-CoV-2 viral lifecycle. SARS-CoV-2 enters host cells by first binding to angiotensin-converting enzyme 2 (ACE2) via the surface spike (S) protein. Following entry of the virus into the host cell, viral genomic RNA is released and translated into viral polymerase proteins. In this process, subgenomic (-) RNAs are synthesized and used as a template to form subgenomic (+) messenger RNAs (mRNAs). The nucleocapsid (N) structural protein and viral RNA are replicated, transcribed, and synthesized in the cytoplasm, whereas other viral structural proteins, including the S protein, membrane (M) protein and envelope (E) protein, are transcribed and then translated in the endoplasmic reticulum (ER). The resulting structural proteins are further assembled into the nucleocapsid and viral envelope at the ER-Golgi intermediate compartment (ERGIC) to form a mature virion, followed by release of the nascent virion from the host cell.

Similar to SARS-CoV and MERS-CoV, the SARS-CoV-2 genome encodes nonstructural proteins (NSPs, such as 3-chymotrypsin-like protease, papain-like protease, helicase, and RNA-dependent RNA polymerase), structural proteins and accessory proteins 22. SARS-CoV-2 has four structural proteins: the spike (S) protein, envelope (E) protein, membrane (M) protein, and nucleocapsid (N) protein. Among these proteins, the trimeric S protein is indispensable for virus-cell receptor interactions during viral entry 23, 24. S protein comprises an N-terminal S1 subunit responsible for virus-receptor binding and a C-terminal S2 subunit responsible for virus-cell membrane fusion. S1 is further divided into an N-terminal domain (NTD) and a receptor-binding domain (RBD) 25, 26. SARS-CoV-2 targets cells through the S protein, which binds to the human angiotensin-converting enzyme 2 (ACE2) receptor and employs the cellular serine protease TMPRSS2 for S protein priming 27-31. Such binding triggers a cascade of events leading to fusion between the cellular and viral membranes for cell entry. The viral RNA genome is released into the cytoplasm after membrane fusion (**see the SARS-CoV-2 lifecycle in Figure 1**). Polyproteins are subsequently synthesized to encode the viral replicase-transcriptase complex. The viral RNA is then synthesized by RNA-dependent RNA polymerase. Synthesis of structural proteins is followed by viral particle assembly and release 32, 33. These viral lifecycle steps provide potential targets for vaccines and therapeutics to prevent and treat SARS-CoV-2 infection. Promising targets include NSPs, which are involved in transcription and replication of the virus, as the key enzymes in the viral lifecycle. Additional targets include viral entry and immune regulation pathways. For example, the S protein plays key roles in the induction of T cell responses and neutralizing antibodies (NAbs), as well as protective immunity, during infection with SARS-CoV-2.

To elucidate the specific mechanisms of SARS-CoV-2 infection, the crystal structure of the SARS-CoV-2 spike RBD bound to the cell receptor ACE2 at 2.45 Å resolution was recently determined 34. The overall binding mode of the SARS-CoV-2 RBD with ACE2 is nearly identical to that of the SARS-CoV RBD, which also utilizes ACE2 on the surface of host cells as a receptor. HeLa cells expressing ACE2 are susceptible to SARS-CoV-2 infection, but cells without ACE2 expression are not infected 1. *In vitro* binding experiments also show that the SARS-CoV-2 RBD has affinity for ACE2 in the low nM range, indicating that the RBD is the key functional component of the S1 subunit responsible for the binding of ACE2 to SARS-CoV-2 34. More structural information at the atomic level would greatly enhance our understanding of the interaction between SARS-CoV-2 and host cells, providing precise targets for NAbs, and assist us in urgently needed structure-based vaccine design in the ongoing fight against SARS-CoV-2. In addition, research on cell modeling tools might promote insight into our understanding of infection mechanisms and speed up the development of therapeutic agents and vaccines for COVID-19 35.

In this review, we aim to discuss the current status of diagnostics, therapeutics, and vaccines against COVID-19. As rapid diagnostics and development of drugs and vaccines for the virus are important interventions to control the outbreak, a review article that encompasses the current research on this topic may provide help for guiding strategies to address the current COVID-19 pandemic.

## Diagnostics

Rapid and accurate detection of COVID-19 is essential to initiate the appropriate treatment rapidly, to limit further spread of the virus and to ultimately eliminate the virus from circulation. COVID-19 patients present a wide range of clinical symptoms (e.g., cough, fever, and dyspnea) that are similar to influenza or other respiratory infections and thus cannot be used for accurate diagnosis 36, 37. At present, accurate and rapid diagnosis has evolved as a tool for detecting this novel coronavirus (**Table 1**). Molecular-based approaches are the first-line methods to confirm suspected cases. Nucleic acid testing is the main technique for laboratory diagnosis. As with other emerging viruses, the development of methods to detect antibodies and viral antigens began after identification of the viral genome. In fact, the genomic sequence of SARS-CoV-2 was released to public databases on January 10, 2020 (GenBank accession No. MN908947), which facilitated the development of standardized laboratory PCR protocols for COVID-19 in January of 2020 38. This was followed by the launch of a range of commercial SARS-CoV-2 PCR-based assays in the last 3 months 39-45.

Reverse transcription quantitative PCR (RT-qPCR) is the most common and straightforward method for the detection of SARS-CoV-2 owing to its advantages as a specific, sensitive and simple quantitative assay, which greatly helps in the diagnosis of early infection. As of May 25, a total of 12 RT-qPCR tests to detect SARS-CoV-2 have been approved in China by the National Medical Products Administration (NMPA) 46. Although RT-qPCR assays using respiratory samples provided sensitive and specific diagnostic tests at the initial phase of the outbreak, these RT-qPCR test kits have many limitations 47. For example, this method is not efficient in rapidly screening a large number of individuals in places where thousands of people transit per hour. In addition, the performance of RT-qPCR depends on many factors, such as the sample type, stage of infection, skill of sample collection, and quality and consistency of the PCR assay being used 48, 49. These problems lead to a noteworthy delay in early diagnosis and subsequent management and pose a serious challenge to providing timely life-support treatment and preventive quarantine. Notably, another powerful and promising tool involves clustered regularly interspaced short palindromic repeats (CRISPR) technology, which is being quickly deployed in the molecular diagnostics landscape. On May 6, Sherlock Biosciences received Emergency Use Authorization (EUA) from the US Food and Drug Administration for its Sherlock™ CRISPR SARS-CoV-2 kit for detection of the virus that causes COVID-19. Based on the SHERLOCK (Specific High-sensitivity Enzymatic Reporter unLOCKing) method, the kit works by programming a CRISPR molecule to detect the presence of a specific genetic signature for SARS-CoV-2 50. If the signature is found, the CRISPR enzyme generates a fluorescent glow. Furthermore, Mammoth Biosciences has developed a rapid (<40 min), easy-to-implement and accurate CRISPR-based assay for the detection of SARS-CoV-2 from respiratory swab RNA extracts called the SARS-CoV-2 DNA Endonuclease-Targeted CRISPR Trans Reporter (DETECTR) 51. CRISPR-based diagnosis methods benefit from high sensitivity and specificity with efficiency and no requirement for elaborate instrumentation. As the assay uses similar specimen collection and nucleic acid extraction methods as the CDC and other RT-qPCR assays, it is subjected to the same potential limitations with regard to the availability of personal protective equipment, extraction kits and reagents. Therefore, many clinicians have proposed that computed tomography (CT) scans should be a necessary auxiliary diagnostic method 52-56. To identify and quarantine patients at an early date, patients clinically diagnosed by chest CT with typical ground-glass lung opacities were also counted as confirmed cases beginning in early February 57-60. However, CT scans also have some shortcomings, such as the hysteresis of abnormal CT imaging and indistinguishability from other viral pneumonia presentations. Accordingly, a combination of chest CT scans and repeated RT-qPCR tests may be helpful for individuals with a high clinical suspicion of SARS-CoV-2 infection but who are negative by RT-qPCR screening. Compared to the currently used nucleic acid detection and CT scans, other methods, such as virus antigen or serological antibody testing, are advantageous with fast turn-around times, high throughput and reduced workload for the detection of novel coronavirus infection. Currently, immunological identification technologies (point-of-care testing (POCT) of IgM/IgG and enzyme-linked immunosorbent assay (ELISA)) kits for SARS-CoV-2 have become available, with detection rates higher than those of nucleic acid detection approaches.

One type of rapid diagnostic test based on host antibody detection has become available more recently 61-64. Antibodies are produced over days to weeks after infection with the virus. Antibody testing is easy to administer and only requires minimal training. It may be helpful as a complement to nucleic acid testing for the diagnosis of suspected COVID-19 cases with negative RT-qPCR results as well as for the identification of asymptomatic infections 65. Confirming suspected cases as early as possible with the benefit of antibody testing might reduce exposure to risk factors during repeated sampling and save valuable RT-qPCR tests. Due to urgency and demand, many antibody test reagents are rapidly being developed and made available on the market to assist in the diagnosis of SARS-CoV-2 infection, including kits for testing total Abs, IgM antibody or IgG antibody, by chemiluminescence, ELISA or colloidal gold methods. On March 6, an anti-SARS-CoV-2 antibody test kit based on chemiluminescent particle immunoassay jointly developed by our team and Beijing Wantai Biological Pharmacy Enterprise Co., Ltd. was approved for listing by the State Drug Administration for emergency approval and is the best performing commercial kit according to the latest WHO validation data 66. The kit is based on the double-antigen sandwich principle that detects total antibodies (including IgM, IgG, IgA and other antibody types). Specific testing for SARS-CoV-2 has great significance for public health and clinical practice, and evidence from studies further demonstrate that RT-qPCR detection combined with serological testing enhances diagnosis sensitivity while maintaining high specificity 67.

Another type of rapid diagnostic test (RDT) that detects the presence of viral antigens expressed by SARS-CoV-2 virus in a respiratory tract sample is of low complexity and may provide results typically within 30 minutes 68,69. The antigen(s) detected are expressed only when the virus is actively replicating; thus, such tests are best used to identify acute or early infection. Monoclonal antibodies (mAbs) against the nucleocapsid protein of SARS-CoV-2 have been developed and might constitute the basis of a future rapid antigen detection test. As of the time of this review, at least 5 antigen-detection RDTs were under evaluation (https://www.finddx.org/covid-19/sarscov2-eval-immuno/), though not all are CE Marking approved or widely available.

## Antiviral therapy

**Figure 2:**
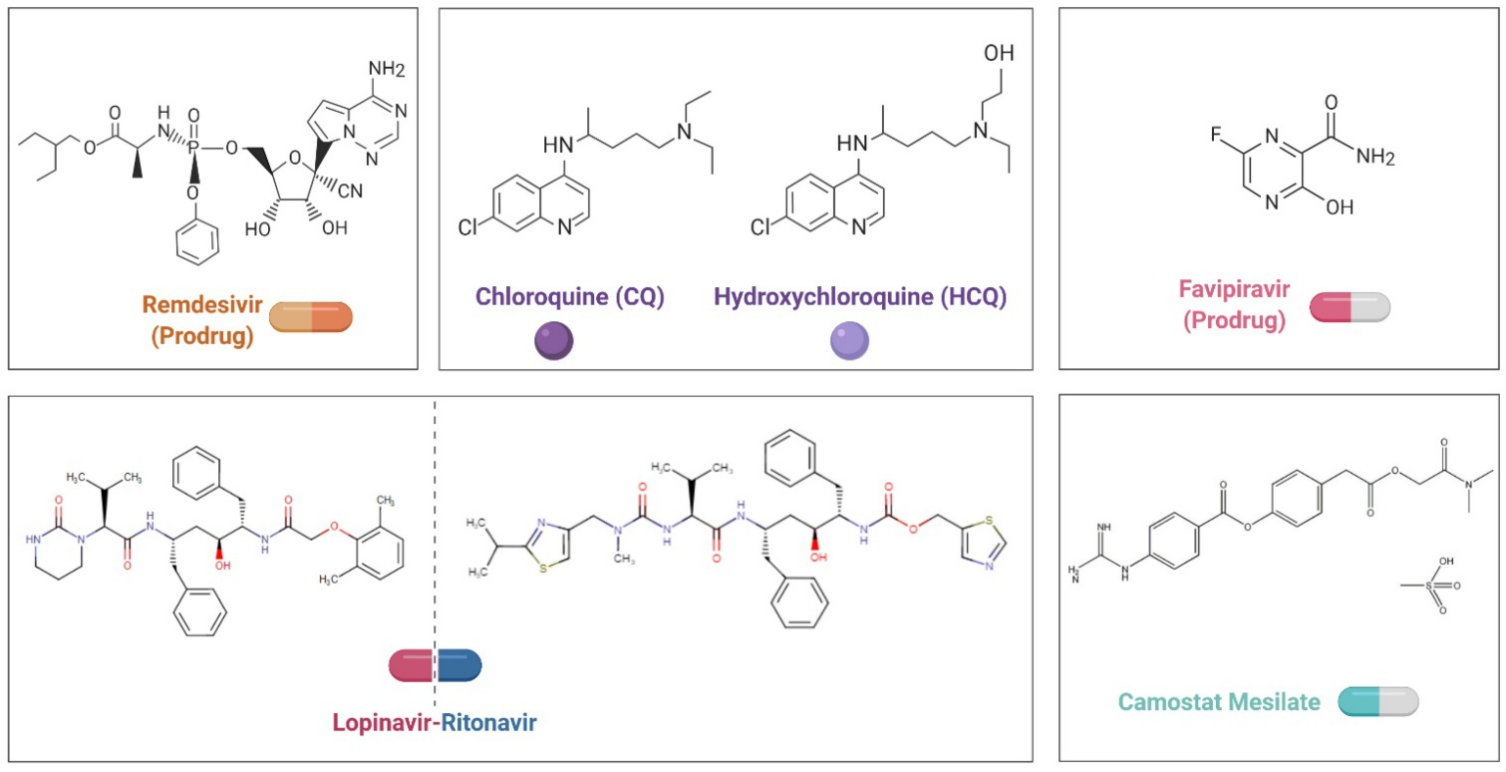
Chemical structural formula of candidate therapeutic agents.

To date, there are no specific antiviral therapies or vaccines for COVID-19 available for human use. Given the urgency of treating these patients, rather than taking months to years to develop and test compounds from scratch, researchers are seeking to repurpose drugs that are already approved for other diseases and have acceptable safety profiles. Small-molecule agents approved for other human diseases can exert their antiviral effect through multiple mechanisms, including blocking viral entry, inhibiting a virally encoded enzyme, targeting a host factor required for replication, or blocking virus particle formation 87. Notably, some antivirals and antimalarials have shown promising abilities to treat patients and reduce the danger of COVID-19 (**Figure 2, Table 2**).

Remdesivir (GS-5734) is a nucleotide analog prodrug that shuts down viral replication by inhibiting a key viral enzyme, RNA polymerase. A recent study reported that remdesivir potently blocks SARS-CoV-2 infection (EC50 = 0.77 μM) in Vero E6 cells 88, and the first patient with confirmed SARS-CoV-2 infection in the United States recovered after receiving intravenous remdesivir in January 89. On April 10, a study reported clinical outcomes for compassionate use of remdesivir in a small cohort of patients with severe COVID-19. Specifically, clinical improvement was observed in 36 of 53 patients (68%) 90. However, a trial (ClinicalTrials.gov: NCT04257656) found that compared with placebo, intravenous remdesivir did not provide significant clinical or antiviral effects in patients with serious COVID-19. Ongoing trials with larger sample sizes will provide information on the efficacy of remdesivir for COVID-19 91. On May 1, the US Food and Drug Administration granted emergency use authorization for remdesivir to treat COVID-19 in adults and children hospitalized with severe disease.

Chloroquine (CQ), a 9-aminoquinoline that is a widely used antimalarial and autoimmune disease drug, has recently been reported as a potential broad-spectrum antiviral drug 92. CQ inhibits virus infection by increasing the endosomal pH required for virus/cell fusion, and a recent study reported its activity against COVID-19 (EC50=1.13 μM) in Vero E6 cells 88, 93. Patients with COVID-19 are being recruited in randomized trials (e.g., ClinicalTrials.gov: NCT04316377, NCT04323527) to determine the safety and efficacy of CQ. Although a few clinical trials have suggested some beneficial effects of CQ for COVID-19, most of the reported data are preliminary 94, 95. Furthermore, it is likely that the risk of adverse reactions to CQ will hamper the successful treatment of patients with COVID-19.

Hydroxychloroquine (HCQ), which exhibits an antiviral effect highly similar to that of chloroquine, might be a better therapeutic approach. Studies have indicated that CQ shows antagonism against SARS-CoV-2 *in vitro*. Chen *et al.* partially confirmed the potential of HCQ in patients with COVID-19 (ClinicalTrials.gov: ChiCTR2000029559) 96. Although positive results from small studies have been reported for hydroxychloroquine, accumulating trial and observational evidence has prompted skepticism regarding whether there are any meaningful clinical benefits for patients with COVID-19. Indeed, a study from France did not support the use of HCQ in patients hospitalized with COVID-19 who require oxygen 97. Additionally, a randomized clinical trial (ClinicalTrials.gov: ChiCTR2000029868) from China shows that hospitalized patients with mild to moderate persistent COVID-19 who received HCQ did not eliminate the virus more quickly than those receiving standard care. Moreover, adverse events were higher among HCQ recipients than nonrecipients 98. Nonetheless, randomized clinical trials are needed to provide definitive causal evidence while offering an opportunity to continuously optimize the treatment plan.

The lopinavir-ritonavir combination, which targets an enzyme that cleaves a long protein chain during the assembly of new viruses, can inhibit HIV protease 99,100. The combination has been evaluated in patients with SARS and MERS, though the results are ambiguous 101-104. Clinical trials (e.g., ClinicalTrials.gov: NCT04255017, NCT04307693) with COVID-19 have been initiated to test the potency of the combination. Recently, a randomized, controlled, open-label trial (ClinicalTrials.gov: ChiCTR2000029308) involving 199 patients with COVID-19 was initiated, but no benefit beyond standard care was observed with lopinavir-ritonavir treatment. Future trials including patients with severe illness may help to confirm or exclude the possibility of a treatment benefit.

Favipiravir (T-705), a purine nucleic acid analog approved for the treatment of influenza, can effectively inhibit the RNA-dependent RNA polymerase (RdRp) of RNA viruses such as influenza, Ebola and norovirus 105. Studies in Vero E6 cells have suggested that favipiravir can cripple the SARS-CoV-2 virus (EC_50_ = 61.88 μM) 88, and patients with COVID-19 are being recruited in randomized trials to evaluate the efficacy of favipiravir plus other antivirals (e.g., ClinicalTrials.gov: ChiCTR2000029600, ChiCTR2000029544).

Camostat mesylate is a potent serine protease inhibitor that has been used primarily to treat pancreatitis and some types of cancer 106. SARS-CoV-2 uses ACE2 for entry and the serine protease TMPRSS2 for S protein priming. Utilizing research on SARS-CoV and the closely related SARS-CoV-2 cell entry mechanism, it has been demonstrated that camostat mesylate can block SARS-CoV-2 infection of lung cells 28. Patients with COVID-19 are being recruited in randomized trials (ClinicalTrials.gov: NCT04374019, NCT04355052) to determine whether this drug reduces SARS-COV-2 viral load in early COVID-19 disease.

## Antibody-based Interventions

The antibody-mediated humoral response plays crucial roles in controlling viral infection. NAbs, which can reduce viral infectivity by binding to the surface epitopes of viral particles and thereby blocking entry of the virus into host cells, are a promising approach to prevent and treat virus infection.

Passive antibody therapy, through transfusion of convalescent plasma from patients who have recovered from viral infections, can also be used as a treatment. Importantly, this therapy offers the only short-term strategy of conferring immediate immunity to infected patients. The antibodies present in convalescent plasma can specifically bind to a given pathogen (e.g., an infectious virus), directly neutralizing its infectivity. Other antibody-mediated pathways, such as antibody-dependent cellular cytotoxicity, complement activation, and/or phagocytosis, may also contribute to their therapeutic effect. There are numerous examples in which convalescent plasma therapy has been successfully applied to treat patients, as follows: SARS; MERS; H1N1 (pandemic 2009 influenza A); H5N1 (avian influenza A); several hemorrhagic fevers, such as Ebola; and other viral infections 116-120. As no specific therapeutic agents or vaccines are available for COVID-19, this therapy is the only strategy that is immediately available for use to prevent and treat a novel, emerging infectious disease such as SARS-CoV-2 infection 121, 122. Shen *et al* reported findings from a preliminary study of 5 severely ill patients with COVID-19 who were given plasma from recovered individuals 123. In this case series, the clinical status of all patients improved by approximately 7 days after transfusion. In addition, the patients' specific NAb titers increased following the transfusion, and respiratory samples became negative within 12 days. Although the limited case size and study design preclude a definitive statement about the potential efficacy of this treatment, the results provide some evidence to support the possibility of evaluating this therapy in more rigorous investigations involving patients with COVID-19.

Moreover, sophisticated technologies are devoted to identifying strongly neutralizing mAbs that can be produced in large quantities. In coronaviruses, the viral component most frequently targeted by NAbs is the surface-located envelope S protein 124-126. To this end, several potently neutralizing human mAbs against SARS-CoV-2 have been developed using various approaches, including single-cell culturing methods of memory B cells isolated from SARS-CoV-2 patients, fusing B cells with an appropriate partner to produce hybridomas, selection of antibodies from phage-display libraries of human antibody fragments, and hybridoma fusion of B cells from immunized transgenic mice encoding human variable immunoglobulin domains 127-131. All of these antibodies were selected based on their *in vitro* neutralizing capacity, and most of them target the SARS-CoV-2 RBD.

Ju *et al.* isolated 206 mAbs specific for the SARS-CoV-2 RBD derived from single B cells of eight SARS-CoV-2-infected individuals 130. Using bioinformatic and biologic characterization, several mAbs with potent binding and neutralizing activity against pseudovirus encoding the S protein and live SARS-CoV-2 have been identified. In particular, the most potent antibodies, P2B-2F6 and P2C-1F11, out-competed ACE2 with close to 100% efficiency, indicating that blocking the RBD and ACE2 interaction is a useful surrogate for antibody neutralization. The results indicate that the humoral immune response is viral species specific and diverse and can produce potent NAbs. Wu *et al.* isolated 4 mAbs specific to SARS-CoV-2 RBD from a convalescent patient 131, and these antibodies effectively neutralized the SARS-CoV-2, with two of them exhibiting additive inhibition effects. Moreover, a therapeutic study in a mouse model validated that these antibodies can reduce virus titers in infected lungs. These antibodies will serve as the most promising candidates for the development of prophylactic and therapeutic interventions against SARS-CoV-2.

CD147-spike protein, a receptor on host cells, is a novel route for SARS-CoV-2 invasion 132. Meplazumab, an anti-CD147 antibody, effectively inhibited SARS-CoV-2 replication and virus-induced cytopathic effects in Vero E6 cells; it also blocks CD147, the receptor of the proinflammatory factor CyPA, thus attenuating inflammation 132,133. Based on this evidence, the team designed an open-label, concurrent controlled trial (NCT04275245) to assess the efficacy and safety of meplazumab in patients with COVID-19 pneumonia. The data demonstrated that meplazumab efficiently improved the recovery of the patients, with a favorable safety profile. However, the results were limited by the absence of a parallel control and a small sample size. Therefore, confirmation from a larger scale and randomized, double-blind trial is required to fully assess the efficacy of meplazumab in patients with COVID-19 pneumonia.

The S proteins of SARS-CoV-2 and SARS-CoV, which are structurally very similar, have an amino acid sequence identity of approximately 77% and commonly bind ACE2 as a host receptor 134-136. To reduce the time that is required to develop SARS-CoV-2-neutralizing antibodies de novo, researchers have assessed available SARS-CoV antibodies for cross-neutralization of SARS-CoV-2, given the high degree of sequence similarity. For example, a SARS-CoV RBD-specific human NAb (CR3022) previously isolated from a convalescent SARS patient is able to bind the SARS-CoV-2 RBD with high affinity (Kd of 6.3 nM) and recognize an epitope on the RBD that does not overlap with the ACE2-binding site 137. However, CR3022 does not neutralize SARS-CoV-2 *in vitro*, and it is possible that this epitope can confer *in vivo* protection 138. Further studies will require suitable animal models, which have yet to be established. Another anti-SARS-CoV antibody, 47D11, was isolated from transgenic H2L2 mice encoding the human immunoglobulin variable regions to generate antibodies that neutralize SARS-CoV-2 (and SARS-CoV). 47D11 binds a conserved epitope on the spike RBD, explaining its ability to cross-neutralize SARS-CoV and SARS-CoV-2, with diverse mechanisms of action that are independent of receptor binding inhibition 139. This antibody offers potential for the prevention and treatment of COVID-19.

Although antibodies are generally beneficial and protective, in rare cases, pathogen-specific antibodies could promote pathology, resulting in a phenomenon known as antibody-dependent enhancement (ADE) 140, 141. The ADE phenomenon is documented for dengue virus and other viruses 142, 143. However, the neutralizing antibodies can be engineered of Fc region with molecular precision to avoid the potential ADE.

## The current pipeline for SARS-CoV-2 vaccines

**Figure 3:**
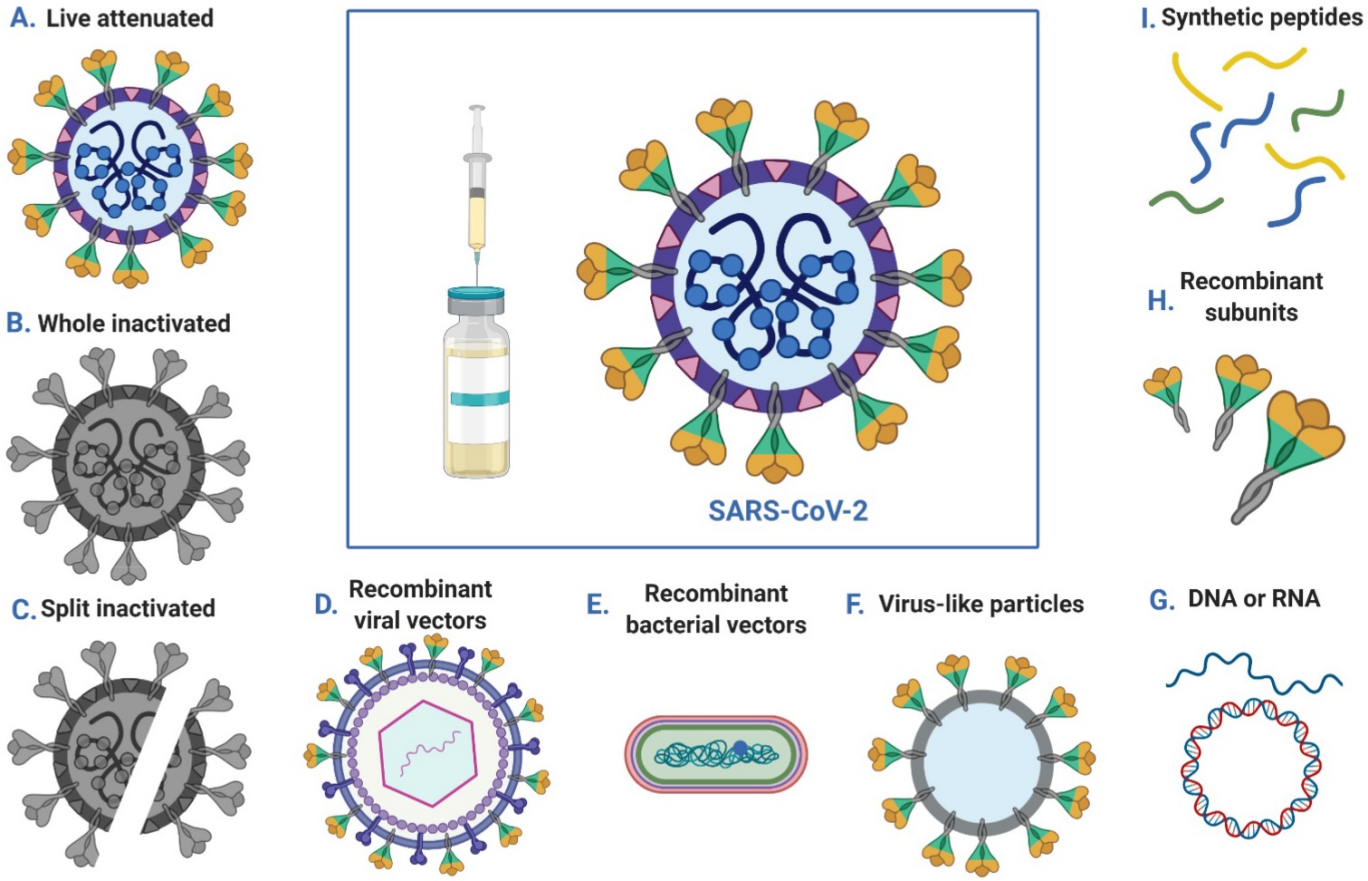
Approaches to SARS-CoV-2 vaccine development.

Vaccination can be used to prevent infection or to reduce disease severity, viral shedding and thereby transmission, thus helping to control SARS-COV-2 outbreaks. Multiple strategies have been employed to generate SARS-COV-2 vaccines, including DNA- and RNA-based vaccines, viral vector vaccines, inactivated virus vaccines, live-attenuated virus vaccines and recombinant protein vaccines (**Figure 3**). According to reports from the WHO, there are more than 100 vaccines against SARS-CoV-2 at various stages of development. Specifically, ten candidate vaccines are already in clinical evaluation (**Table 3**).

**mRNA vaccines** are an emerging new vaccine modality in which patients are treated with mRNA oligonucleotides that encode either an antigen of interest or an antigen as well as its viral replication machinery 144. The potential advantages of the mRNA approach to prophylactic vaccines include the ability to mimic natural infection to stimulate a more potent immune response, combining multiple mRNAs into a single vaccine, rapid discovery to respond to emerging pandemic threats and manufacturing agility derived from the platform nature of mRNA vaccine design and production 145. mRNA vaccines have elicited potent immunity against infectious disease targets in animal models of infection with influenza virus, Zika virus, rabies virus and others, especially in recent years, using lipid-encapsulated or naked forms of sequence-optimized mRNA 146-148. Moderna and the Vaccine Research Center, a unit of the National Institute of Allergy and Infectious Diseases (NIAID), have collaborated to develop mRNA vaccines (mRNA-1273) for the novel coronavirus. mRNA-1273 encodes a prefusion stabilized form of the SARS-CoV-2 S protein. The vaccine consists of a mRNA drug substance that is manufactured into lipid nanoparticles (LNPs) composed of the proprietary ionizable lipid SM-102 and 3 commercially available lipids: cholesterol, DSPC, and PEG2000 DMG. Extensive basic research into RNA and lipid and polymer biochemistry has enabled translating mRNA vaccines into clinical trials. On May 18, Moderna announced positive interim phase 1 results for mRNA-1273 against SARS-CoV-2 149. After two doses, mRNA-1273 elicited neutralizing antibody titer levels at or above the levels in convalescent sera in all initial participants. To continue progress on this potential vaccine during the ongoing global public health emergency, Moderna intends to work with the FDA and other government and nongovernment organizations to be ready for phase 2 and any subsequent trials, which are anticipated to include a large number of subjects and will seek to generate additional safety and immunogenicity data. Furthermore, BioNTech and Pfizer are jointly developing another mRNA vaccine (BNT162), which has been approved for a phase 1/2 clinical trial to determine the optimal dose for further studies as well as to evaluate the safety and immunogenicity of the vaccine. The trial is the first clinical trial of a COVID-19 vaccine candidate to start in Germany and is part of a global development program.

**DNA vaccines** are appropriate for emerging infectious diseases because they allow for the rapid design of multiple candidates for novel antigens 150. In addition, they are directly injected or otherwise inoculated into recipients. Recently, various DNA vaccine platforms have been developed to improve the efficacy of vaccines by using electroporation (EP) to deliver plasmids and adding adjuvants to enhance immune responses. Inovio Pharmaceuticals was the first to advance its vaccine (INO-4700) against MERS-CoV 151, a related coronavirus, into evaluation in humans, and this company has developed a DNA vaccine (INO-4800) to prevent infection by SARS-CoV-2. INO-4800 induces T cell activation by delivering DNA plasmids that express the SARS-CoV-2 S protein. The effects based on measuring functional antibodies and antigen-specific T cell responses in multiple animal models are particularly encouraging 152. Inovio has also initiated an open-label trial (NCT04336410) to evaluate the safety, tolerability and immunological profile of INO-4800 administered by intradermal (ID) injection followed by EP using a CELLECTRA® 2000 device in healthy adult volunteers.

**Viral vector vaccines**, which function as viral gene expression and delivery systems, rely on a host viral genome, and an unrelated viral genome lacking packaging elements is engineered to encode antigenic components of the virus of interest to elicit an immune response. Because viral vector vaccines persist in the host as genetic material, they directly infect antigen-presenting cells and have strong immunogenicity, efficiently inducing B cell- and T cell-mediated immune responses. Furthermore, viral vector vaccines can result in high-titer NAbs. Several different virus vectors have been developed as SARS-CoV-2 vaccines. CanSinoBIO and the Beijing Institute of Biotechnology (BIB) have collaborated to develop an adenovirus type 5 vector-based novel coronavirus vaccine (“Ad5-nCoV”) that encodes the full-length S protein of SARS-CoV-2. Ad5-nCoV protects against SARS-COV-2 infection by relying on a recombinant replication-defective human adenovirus type-5 vector to induce the immune response. The vaccine candidate is built upon CanSinoBIO's adenovirus-based viral vector vaccine technology platform, which has also been successfully applied to develop a globally innovative vaccine against Ebola virus infection. The results from preclinical studies of Ad5-nCoV show that the vaccine candidate can induce a strong immune response in animal models, and preclinical animal safety studies demonstrate a good safety profile. This is currently the first novel coronavirus vaccine for COVID-19 that has progressed to this stage in China. A first-in-human trial showed that the Ad5-nCoV vaccine is tolerable and immunogenic in healthy adults 153, with specific humoral responses against SARS-CoV-2 peaking at day 28 postvaccination and rapid specific T-cell responses noted at day 14. There is potential for further investigation of the Ad5-nCoV vaccine for the control of the COVID-19 outbreak. Furthermore, University of Oxford researchers have begun testing a new COVID-19 vaccine called ChAdOx1 nCoV-19; it is derived from a virus (ChAdOx1) that is an attenuated version of a common cold virus (adenovirus) causing infections in chimpanzees, but it has been genetically modified such that it is unable to proliferate in humans. The vaccine is based on an adenovirus vector (ChAdOx1) and the SARS-CoV-2 S protein. The results showed that a single vaccination with ChAdOx1 nCoV-19 is effective for preventing lung damage upon high-dose challenge with SARS-CoV-2; however, all of the monkeys vaccinated with ChAdOx1 nCoV-19 became infected when challenged, and there was no difference in the amount of viral RNA detected from nasal secretions in the vaccinated and unvaccinated monkeys 154. As of May 13, more than 1,000 volunteers participated in the trial, which aims to assess whether healthy people can be protected from COVID-19. It will also provide valuable information on the safety aspects of the vaccine and its ability to generate good immune responses against the virus. In addition to adenovirus, our team and the University of Hong Kong have used influenza virus vectors for the development of candidate SARS-CoV-2 vaccines that are in preclinical phases.

**Inactivated virus vaccines** use chemicals (such as formalin, β-propiolactone and diethylpyrocarbonate) or heat to render the viral genome noninfectious while maintaining the virion structure, thus preserving antigenicity but eliminating the potential to cause productive infection. Thus, in theory, inactivated virus vaccines are easily prepared and antigenically similar to live viruses. Such vaccines have been found to be effective and safe for the prevention of diseases caused by viruses such as poliovirus and influenza virus 155, 156. Several institutes are developing inactivated virus COVID-19 vaccines. Recently, the Beijing Institute of Biological Products and Wuhan Institute of Biological Products have developed an inactivated novel coronavirus pneumonia vaccine. Healthy people at different ages are being recruited in randomized, double-blind, placebo parallel-controlled phase I/II clinical trials (ClinicalTrials.gov: ChiCTR2000032459, ChiCTR2000031809) to explore the safety and immunogenicity of this COVID-19 vaccine. Drawing on previous experience in SARS vaccine development, Sinovac has developed another inactivated vaccine. On April 13, 2020, a randomized, double-blinded, placebo-controlled phase I clinical trial (ClinicalTrials.gov: NCT04352608) of an inactivated SARS-CoV-2 vaccine was approved by China's NMPA to evaluate the safety and preliminary immunogenicity in healthy adults, with two different dosages of the vaccine candidate 157.

**Other vaccine approaches** include live-attenuated vaccines that are produced by reducing or eliminating the virulence of a live virus, typically using chemical-driven or site-directed mutagenesis. Thus, the virus is capable of productive infection, but the resulting disease is either diminished or eliminated. Live-attenuated vaccines can elicit both innate and adaptive immune responses, and protection can be life-long. Additionally, their production is inexpensive. Codagenix and the Serum Institute of India are using viral deoptimization to synthesize "rationally designed", live-attenuated vaccines against the emergent coronavirus. Recombinant-protein-based vaccines are advantageous over other types of vaccines in that they are safe and have few side effects, inducing the immune system without the introduction of infectious viruses. One example is the NVX-CoV2373 vaccine developed by Novavax, which is in the clinical stage 158. It was created using recombinant nanoparticle technology to generate antigen derived from the S protein and contains Matrix-M™ adjuvant to enhance the immune response and stimulate high levels of neutralizing antibodies. A trial of Clover's S-trimer vaccine developed by Clover Biopharmaceuticals will be recruiting healthy adults in Australia, and this vaccine will be widely available upon confirmation of its safety and efficacy. In addition, LV-SMENP-DC and pathogen-specific aAPC vaccines from Shenzhen Geno-Immune Medical Institute are in phase 1 clinical trials to evaluate safety and immune reactivity 159,160.

## Conclusions and Prospects

Despite tremendous efforts to prevent the spread of SARS-CoV-2 worldwide, the high mortality and person-to-person transmission pose a significant threat to global public health. Diagnostics are important for dealing with virus outbreaks because they can enable healthcare workers to direct efforts to patients with COVID-19. Remarkably, various diagnostic kits to test for COVID-19 are available. Several drugs have demonstrated *in vitro* activity against the SARS-CoV-2 virus or potential clinical benefits in observational or small, nonrandomized studies. Additionally, researchers are evaluating several different technologies, and more than 100 vaccines are under development against SARS-CoV-2 worldwide. Specifically, ten candidate vaccines have already been administered to volunteers in safety trials; others have started testing in animals. However, as the development of vaccines for human use can take years, their development will likely come too late to impact the first wave of the pandemic. These challenges will remain in the long term. Clearly, there is an urgent need for effective antiviral therapy and vaccines for this emerging global threat.

There are a series of patient-associated and virologic factors that pose major clinical challenges to the development of anti-SARS-CoV-2 drugs. SARS-CoV-2 is a virus with diversity that becomes mutated, and novel SARS-CoV-2 mutants are likely to emerge repeatedly at unpredictable times 161,162. Therefore, most drugs that specifically target the existing SARS-CoV-2 may not be effective against a mutant SARS-CoV-2. The lag time between mutant emergence and the development of new prophylactic treatments is of concern. Thus, there is an urgent need for the development of new, broad-spectrum drugs that target conserved sites to prepare for future outbreaks involving mutants. Recent studies have reported that lipopeptide EK1C4 shows exceptional promise to be developed as the first pan-CoV fusion inhibitor-based antiviral therapeutic or prophylactic for treatment or prevention of infection by the currently circulating SARS-CoV-2 and mutants or emerging SARS-related CoVs 163-165. In addition, combining the advanced knowledge of bioinformatics and immunology to screen and confirm the conserved dominant epitopes of SARS-CoV-2, it will be helpful to develop therapeutic antibodies with broad-spectrum neutralization activity and vaccines that can induce a broad-spectrum antiviral immune response. Currently, there are a limited number of animal models available for infections caused by SARS-CoV-2. Normally, antiviral therapy or vaccines enter into human trials after tests for safety and effectiveness in appropriate animal models. However, the virus does not grow in wild-type mice and induces only mild disease in transgenic animals expressing human ACE2 166,167. The limited availability of mouse-adapted virus strains and ACE2 transgenic mice remains a major obstacle to testing anti-SARS-CoV-2 drugs or vaccines. Other potential animal models include ferrets and nonhuman primates (NHPs), for which pathogenicity studies are ongoing. In addition, understanding how the immune system interacts with the pathogen and the vaccine itself is crucial for avoiding pitfalls, such as ADE phenomenon, a process in which a virus leverages antibodies to promote infection.

The viral load curve of COVID-19 is similar to that of influenza, and the viral load is very high at the time of presentation 168,169. Experience from the treatment of influenza patients, in this case of high-viral load patients, suggested that the combination of multiple antiviral drugs is more effective than single drug treatments 170-172. Indeed, the combination of appropriate antiviral drugs might rapidly suppress the high initial viral load, improve the clinical parameters, and reduce the risk to medical staff by controlling the spread of the virus. A multicenter randomized open-label phase 2 trial (ChiCTR2000029308) showed that triple antiviral therapy with interferon beta-1b, lopinavir-ritonavir, and ribavirin was safe and superior to lopinavir-ritonavir alone in alleviating symptoms, suppressing shedding of SARS-CoV-2, and facilitating discharge of patients with mild to moderate COVID-19 168. There are also many other studies investigating combination therapy; these smaller clinical studies examining virologic endpoints may be sufficient to identify which combinations can be used for large studies in risk groups or hospitalized patients. For combinations involving immunomodulatory interventions, the challenges may be great because the goal is disease amelioration by regulating the host complex responses. Regardless, it is certain that as our continuous in-depth understanding of the immune response dynamics and the disease pathogenesis of SARS-CoV-2 deepens, combination therapy involving immunomodulation, vaccines or other immunotherapies will have great potential.

The outbreak has emphasized the urgent need for renewed efforts to develop specific antiviral therapies or vaccines to combat SARS-CoV-2. We need well-developed emergency plans that allow us to develop, test, produce, and distribute agents or vaccines as quickly as possible. Such endeavors would require tight coordination among governments, regulatory agencies, pharmaceutical companies, and the WHO, as well as novel approaches and clinical trial design.

## Figures and Tables

**Table 1:** Diagnostics methods for COVID-19

Method	Characteristics	Limitations	Refs
CT Scan	Available earlier; check severity of condition; check possible infection	Expensive; unable to distinguish from other viral pneumonias; hysteresis of abnormal CT imaging	52-60, 70-72
RT-qPCR	Specific, sensitive and simple quantitative assay, which greatly helps in the diagnosis of early infection	Costly and time consuming to perform; more prone to false negatives or low value	38-42, 70, 73-75
CRISPR-based detection	High sensitivity and specificity with efficiency and no requirement for elaborate instrumentation	Certain biological safety hazards brought by the retention and operation of patient samples	50, 51, 76-78
Antibody-detection	Fast, robust and easy to perform; requiring only a small amount of sample	Unable to detect the presence of infection during the early stage of disease; cross-reactivity	61-64,79-82
Antigen-detection	Low complexity; rapid; easy to perform	Best used to identify acute or early infection; more prone to false negatives	68, 69,83-86

**Table 2:** List of candidate therapeutic agents for COVID-19 treatment

Candidate therapeutic	Manufacturer	Target	Mechanism of action	Infectious diseases	Status of clinical trials	Refs.
Remdesivir (GS-5734)	Gilead	RdRp	Terminates the nonobligate chain	Ebola, SARS-CoV-2	• Phase 2 | phase 3 for Ebola (NCT03719586) • Phase 3 for SARS-CoV-2 (NCT04292899, NCT04292730, NCT04280705, NCT04321616, etc.)	88-91, 107, 108
Chloroquine (CQ)	Sanofi	Endosomal acidification	A lysosomotropic base that appears to disrupt intracellular trafficking and viral fusion events	SARS-CoV, MERS-CoV, SARS-CoV-2	•Approved for malaria and certain amoeba infections • Under clinical trial for SARS-CoV-2 Phase 2 (NCT04323527, NCT04328493, NCT04344951, NCT04342650, etc.) Phase 3 (NCT04324463, NCT04341727, etc.) Phase 4 (NCT04316377 NCT04362332, etc.)	92, 104, 109
Hydroxychloroquine (HCQ)	Sanofi	Endosomal acidification	Hydroxychloroquine shares the same mechanism of action as chloroquine	SARS-CoV, MERS-CoV, SARS-CoV-2	Under clinical trial for SARS-CoV-2 Phase 2 (NCT04329832, NCT04335552, etc.) Phase 3 (NCT04345692, NCT04342221, etc.) Phase 4 (NCT04362332, NCT04355026, etc.)	110-112
Lopinavir-ritonavir	Abbott	3CL protease	Inhibits 3CLpro	HIV, SARS-CoV-2	• Under clinical trials for SARS • Under clinical trial for SARS-CoV-2 Phase 2 (NCT04307693, NCT04328012, etc.) Phase 3 (NCT04328285, NCT04364022, etc.) Phase 4 (NCT04255017, NCT04350684, etc.)	99, 103, 104
Favipiravir (T-705)	Toyama	RdRp	Inhibits RdRp	Influenza, SARS-CoV-2	• Approved for influenza in Japan • Under clinical trial for SARS-CoV-2 Phase 2 (NCT04358549, NCT04351295, etc.) Phase 3 (NCT04349241, NCT04303299, etc.) Phase 4 (NCT04359615)	105, 113-115
Camostat mesilate (Foipan^TM^)	Ono Pharmaceutical	TMPRSS2	Inhibits serine protease	SARS-CoV-2	Under clinical trial for SARS-CoV-2 Phase 2 (NCT04374019) Phase 3 (NCT04355052)	106

**Table 3:** Clinical-phase vaccine candidates for COVID-19

Vaccine strategy	Candidate	Lead developer	Vaccine characteristics	Status	Same platform for noncoronavirus candidates
RNA vaccines	mRNA-1273	Moderna/NIAID	Lipid nanoparticles containing the mRNA vaccine encoding the SARS-CoV-2 S protein	Phase 2 (IND accepted) Phase 1 NCT04283461	Multiple candidates
	BNT162	BioNTech/Fosun Pharma/Pfizer	Lipid nanoparticles containing the mRNA vaccine encoding the SARS-CoV-2 S protein	Phase 1/22020-001038-36 NCT04368728	-
Viral vector-based vaccines	Ad5-nCoV	CanSino Biological Inc./Beijing Institute of Biotechnology	Nonreplicating adenovirus type 5 vector carrying the gene for the SARS-CoV-2 S protein	Phase 2 ChiCTR2000031781, phase 1 ChiCTR2000030906	Ebola
	ChAdOx1 nCoV-19	University of Oxford	ChAdOx1 construct (an adenovirus vaccine vector) carrying the gene for the SARS-CoV-2 S protein	Phase 1/2 NCT04324606	MERS, influenza, TB, Chikungunya, Zika, MenB, plague
DNA vaccines	INO-4800	Inovio Pharmaceuticals	DNA plasmid encoding the SARS-CoV-2 S protein delivered by electroporation	Phase 1 NCT04336410	Lassa, Nipah, HIV, filovirus, HPV, cancer indications, Zika, hepatitis B
Inactivated virus vaccines	Inactivated	Beijing Institute of Biological Products/Wuhan Institute of Biological Products	Virions inactivated with chemicals or radiation, with or without adjuvant, rendering the viral genome noninfectious while maintaining the virion structure, which is antigenically similar to the live virus	Phase 1/2 ChiCTR2000032459	-
	Inactivated	Wuhan Institute of Biological Products/Sinopharm		Phase 1/2 ChiCTR2000031809	-
	Inactivated + alum	Sinovac		Phase 1/2 NCT04383574 NCT04352608	SARS
	Inactivated	Institute of Medical Biology, Chinese Academy of Medical Sciences		Phase 1	-
Protein subunit	NVX-CoV2373	Novavax	Full length recombinant SARS-CoV-2 glycoprotein nanoparticle vaccine adjuvanted with Martrix M^TM^ adjuvant	Phase 1/2 NCT04368988	RSV, CCHF, HPV, VZV, EBOV

Source: WHO.
